
JOURNAL INFORMATION
==============================
NLM Title Abbreviation: J Can Health Libr Assoc
Journal ID: J Can Health Libr Assoc
NLM Journal ID: 101229936
Journal ID: JCHLA

The Journal of the Canadian Health Libraries Association / Journal de l'Association des bibliothèques de la santé du Canada

EISSN: 1708-6892
Publisher: Canadian Health Libraries Association / Association des bibliotèques de la santé du Canada

ARTICLE INFORMATION
==============================
PMCID: PMC9359090
DOI: 10.29173/jchla29625
Article ID: JCHLA-43-068
Article version: 1
Subjects: Abstracts

CHLA 2022 CONFERENCE ABSTRACTS / RÉSUMÉS DU CONGRÈS ABSC/CHLA 2022

Electronic publication date: 2022 Aug 4
Publication date: 2022 Aug
Volume: 43
Issue: 2
First page: 68
Last page: 91
License: This article is distributed under a Creative Commons Attribution License: https://creativecommons.org/licenses/by/4.0/
License URL: https://creativecommons.org/licenses/by/4.0/

==============================

IW = Interactive Workshop

### IW1. Practical Guidance to Finding the Best Evidence During Public Health Emergencies

This workshop will provide health science librarians and information professionals at any level/context with an overview of the best practices in finding and identifying the best scientific evidence during novel public health emergencies. Attendees will be presented with an overview of a best practices statement developed by the Librarian Reserve Corps. Attendees will then apply the recommendations from the best practices statement in designing a plan to respond to real-life case study/information request during a public health emergency. A discussion period will follow on how to apply the best practices in other contexts, environments, and cultures. Attendees will also be invited to share their own experiences and best practices during the discussion session. Through hands-on learning and discussion, librarians and information professionals at any level/context will develop strategies to find and critically appraise the best evidence in any novel public health emergency situation.

CP= Contributed Papers

### CP1. The Right Tool, at the Right Time, for the Right Task: Information Management Platforms Used During the COVID-19 Pandemic

Minakshi Sharma[1], Jeff Maus[1], Taiqi Ran[1], Kelly Sabaliauskas[1], Jielan Xu[2] Sabrena Yang[1], Michael Young[3] & Toronto Public Health Web Services Team[1]
Toronto Public Health[1], City of Toronto[2] & Toronto Paramedic Services[3]

**Introduction:** During the pandemic, public health organizations required agile information systems. Keeping staff up to date, monitoring the health of the larger community and ensuring adherence to changing regulations required the use of nimble platforms while addressing concerns around access, privacy, and information security. **Description:** Information management was made possible by using LibGuides, intranet sites, online survey tools, Moodle, and email blasts. We will outline the features of each platform and its suitability to the required task. First, the LibGuides platform was used to collate early evidence that emerged on SARS-CoV-2 and offer the city a space to gather information on municipal recovery plans. Second, as provincial or national guidelines changed during the COVID-19 pandemic, information management tools were set up to help the COVID-19 case and contact management teams manage the flow of information, generate thousands of case and contact letters and emails, and stay abreast of constantly changing circumstances. Third, survey tools were utilized to collect information from the public and various Health Inspectors in the field. Fourth, the Moodle learning management platform enabled the mass immunization clinics to share information with both internal and external staff recruited for the city's nine vaccination clinics. Finally, a COVID-19 weekly digest highlighted the latest published or grey literature. **Outcomes:** Available technology or content management platforms were used in novel ways during the pandemic. We will share advantages, limitations, user feedback, and lessons learned. **Discussion:** By reflecting on the pandemic, we share insights on how information can be appropriately collected, packaged or presented.

### CP2. From Silos to Synergy: Engaging in Interprofessional Collaboration during the COVID-19 Pandemic to Co-Create Information Dissemination Portals

Minakshi Sharma[1], Jill Cheyne[2], Ashley Corallo[1], Tracey Dal Bianco[1], Julia Dearing-Vollett[1], Ann Liddy[1], Chloe Pacht[1], Taiqi Ran[1], Marisa Seto[1], Toronto Public Health Web Services Team[1] & Michael Young[3]
Toronto Public Health[1], City of Toronto[2] & Toronto Paramedic Services[3]

**Introduction:** The COVID-19 pandemic compelled public health organizations to undergo significant transformation and rethink key elements of their business processes. Remote work meant heavier reliance on technical skills and information management (IM) platforms to support COVID-19 Community Case and Contact Management (CC&CM), Mass Immunization Clinics (MICs) or Lab Liaison (LL) work. **Description:** Toronto Public Health (TPH) Library Staff engaged in three IM initiatives. First, library staff collaborated with Web Services Staff and Knowledge Translation (KT) leads to design, develop and maintain the CC&CM Intranet Site with real-time updates communicating changes to processes, FAQs, workflows, “How To” documents and guidelines. Next, library staff collaborated with Paramedic Services Staff and Health Policy Specialists to launch the MICs Staff Information Portal which provided internal and external staff with the resources required across 9 clinics. Finally, lab redactions were undertaken in collaboration with Lab Liaisons to streamline access to lab related resources required by public health staff. **Outcomes:** Public health staff were breaking down silos, leveraging technology and engaging in interprofessional collaboration (IPC). IPC year has enabled KT and successful implementation of IM projects. Effective use of technology ensured frontline staff saved time and accessed the most up to date resources such as guidelines, protocols or directives. Based on staff feedback and team requirements, the information portals have undergone changes over the last year. **Discussion:** What information is needed by frontline staff for effective and efficient professional practice and how can library, KT, and IM skills be implemented in future pandemics?

### CP3. Experiences of Public Health Library Staff During the COVID-19 Pandemic: Stepping Up to Support Our Organizations and Reflecting on Lessons Learned

Amy Faulkner[1], Minakshi Sharma[2],Maria Aulicino[3] & Tanya Harron[4]
Simcoe Muskoka District Health Unit[1], Toronto Public Health[2], The Regional Municipality of York[3] & Wellington-Dufferin-Guelph Public Health

**Introduction:** The Government of Ontario declared its first provincial emergency in response to COVID-19 in March 2020. All health organizations, especially public health units, were at the forefront of the pandemic response. Almost two years later, the public health professionals at Ontario's Public Health Units (PHUs) continue to adjust and adapt to the ever changing municipal, provincial, and federal guidelines, protocols, and orders. Public health information professionals supported the work of their respective PHUs in various ways. **Description:** Using programs, projects and activities from across the province we provide a snapshot of pandemic related library and information supports. Library staff were aggregating research resources, fielding queries about various aspects of SARS-CoV-2 or COVID-19 (transmission, epidemiology, vaccine hesitancy, etc.), providing copyright support, engaging in knowledge translation activities and collaborating across organizations. **Outcomes:** Personal and collective reflections will be shared on how to improve pandemic-related information products or supports. We plan to share usage statistics (e.g. LibGuides or website statistics). We will share lessons learned across PHUs with respect to information products and services. **Discussion:** What role did library staff play? What lessons were learned about information access and transferability of library skills to pandemic-related activities?

## CP4. Urgent, Important, and Due ASAP: Providing Public Health Library Services in a Global Pandemic

Beata Pach, Allison McArthur, Domna Kapetanos, Hannah Skinner, Lindsay Harker & Susan Massarella
Public Health Ontario

**Introduction:** Our library is situated within a provincial public health agency with a mandate to generate and share public health evidence and technical advice. The Library Services (LS) team is embedded in the process of developing knowledge products and plays an active role supporting the COVID-19 pandemic response. **Description:** Building on lessons learned from H1N1, LS began proactively disseminating information on COVID-19 even before the pandemic was officially declared, delivering daily summaries of peer-reviewed literature and preprints on COVID-19 to staff. A second daily scan of news and guidelines from public health organizations was also shared internally and on the website. As the body of scientific evidence grew, LS implemented search alerts, topic collections, and ad-hoc searches on hot topics related to COVID-19. **Outcomes:** LS conducted ~1400 literature searches in support of most of the COVID-19 knowledge products delivered. Since new rapid review-style publications were established to keep up with the proliferation of COVID-19 evidence, LS is tasked with reviewing them to ensure the correct, complete, and consistent citation of scientific sources. We also extended library support to stakeholders such as the COVID-19 science advisory groups. **Discussion:** To meet the increased demand for and urgency of library requests during the pandemic, LS created a triage and task sharing protocol to ensure that the quality and comprehensiveness of the literature searches was maintained. We also partnered with librarian colleagues at the local level. LS's collaboration with health units increased public health system efficiency by avoiding duplication of effort among stakeholders.

## CP5. Reduce, Reuse, Repository: Creating a Hospital Poster Repository to Mitigate Research Waste, Mobilize and Preserve Institutional Knowledge

Zack Osborne, Michael Myers, Teruko Kishibe
Unity Health Toronto

**Introduction:** Posters are a popular format to present and share research. However, research output in poster formats in the biomedical and health sciences (e.g. in medical education, healthcare quality improvement initiatives, etc.) are commonly lost or forgotten because they are not preserved, catalogued, indexed, or discoverable in many storage and retrieval databases. This loss represents a significant waste of time, labour, resources, applied research, and new knowledge. **Description:** Identifying this gap, the Unity Health Toronto Library Services and Archives team undertook a project to establish a repository system to preserve, describe, and make accessible the valuable local research output by hospital staff, students, researchers, and physicians. Further, this initiative aligns with new opportunities to capture poster research created in the pandemic-era during a time when conference poster presentations pivoted exclusively to online. Building a hospital institutional repository for academic, scholarly, education, and scientific posters standardizes description, enables access and discovery of past project outcomes and research initiatives, and supports poster authors to share and mobilize the knowledge research teams have created. **Outcomes:** This presentation will outline the motivations, approach, systems and tools, and considerations applied to build a research poster repository. The repository is planned to launch its initial phase in the spring of 2022. **Discussion:** An overview and discussion of its successes, challenges, and next steps will be shared at the time this presentation is delivered.

## CP6. Novel Coronavirus, Novel Ideas: Collaborative Creation of a Mandatory Information Literacy Module for First-Year Health Sciences Students

Lydia Thorne
Ontario Tech University

**Introduction:** As post-secondary institutions continue to offer more online and hybrid courses in response to the COVID-19 pandemic, addressing virtual learners' information literacy needs has become more important than ever before. When the university abruptly moved all classes online in March 2020, our team was able to quickly pivot and create a Canvas module to replace face-to-face instruction in a core undergraduate course**. Description:** All first-year health sciences students are required to take a common subject, namely Information Literacy and Written Communication. Content for the module was developed in collaboration with instructors in order to meet course and assignment requirements. The combination of text, videos, and activities introduce students to key topics, from choosing an appropriate topic to critically evaluating sources. Students complete quizzes throughout the module that contribute to a percentage of their final course grade. **Outcomes:** Since the module was first launched in Fall 2020, it has been used in all 14 sections of this course and completed by over 1,100 students. Feedback collected from user satisfaction surveys has been very positive to date. Students reported that the module was challenging but engaging and that its self-paced, interactive nature enhanced their learning. **Discussion:** An online module has allowed us to provide consistent, baseline information literacy instruction to all first-year students in the Faculty of Health Sciences. We will highlight some best practices, lessons learned, and future plans for this project in light of the pandemic.

## CP7. Information Seeking in the Time of COVID: How Do Young Adults Find COVID Related Information?

Joan C. Bartlett, Aaron Bowen-Ziecheck & Sofie Tsatas
McGill University

**Introduction:** Past research indicates that young adults rely heavily on information obtained from the web and social networks, but also that they may not be able to judge the authenticity, validity, and reliability of the digital information, and may share misinformation among themselves. Our prior work found inconsistencies between the resources judged most credible for health information, and the resources used most frequently. Credible resources were reported to be inaccessible, with reliance instead on Internet (including social media) and personal sources. Librarians were not generally considered as "experts" with respect to health information. As part of this ongoing program of research into young adults' information behaviour and information literacy, the current study investigates health related information seeking and use, specifically in the context of the COVID-19 pandemic. **Methods:** Data collection uses semi-structured interviews with McGill University undergraduate students. Questions include which resources are used most frequently to find COVID-19 related health information, how and why credibility is judged, and how and why information resources are selected. The interviews also discuss navigating fake news and misinformation. We anticipate reaching data saturation with 15-25 participants. **Results:** Data collection and analysis are in progress; preliminary results will be presented. **Discussion:** We will discuss the implications of the results for libraries, librarians, and other information service providers, with attention to information literacy, consumer health information services, and the challenges of mitigating fake news and misinformation.

## CP8. Searches as Data: Using an Institutional Data Repository to Archive and Share Knowledge Synthesis Searches

Jill T. Boruff & Alisa B. Rod
McGill University

**Introduction:** With the introduction of PRISMA-S, librarians need a way to archive and share detailed search histories that does not rely exclusively on journal publishers and allows librarians to remain in control of this important research output. By defining search strategies and related database exports as code or scripts and data, we can expand the mandate of the data management infrastructure to include this work. The objective of this project was to create a space in our institutional data repository for our librarians to deposit and share their search strategies for knowledge syntheses (KS). **Description:** A health sciences librarian and the research data management specialist created a “KS Search Materials Repository” in McGill University's Dataverse. They hosted a half-day "data-thon" where they worked with the health sciences librarians to develop a standardized KS data management plan, search reporting documentation, and how-to guidance for the repository. **Outcomes:** Six librarians are now trained to deposit KS searches and seven sub-repositories were created. We will use Dataverse to track the number of searches conducted and the impact of the deposited search strategies (via download numbers and citations). We published the documentation under a CC-BY license to encourage broader implementation of this search sharing workflow. **Discussion:** In addition to better documentation and tracking of KS searches at our institution, the Dataverse enables sharing of searches among colleagues with discoverable metadata fields for searching within and across deposited searches. Since customizable instances of Scholars Portal Dataverse are available to most Canadian institutions, this model could be adopted by librarians across Canada.

## CP9. Successes and Challenges of a 3 Year-Long Research Project: How We Did It and You Can Too

Glyneva Bradley-Ridout, Erica Nekolaichuk & Elena Springall
University of Toronto

**Introduction:** In July 2021, we published a research study titled "UpToDate versus DynaMed: a cross-sectional study comparing the speed and accuracy of two point-of-care information tools", marking the end of a multi-year journey. A single publication belies the years of work that goes into planning and conducting a research project. In this presentation, we will share a “behind-the-scenes" glimpse into our experiences executing our research project, including our challenges, successes, and lessons learned. **Description:** Health Sciences Libraries are often faced with difficult budget decisions regarding which clinical tools to purchase, while keeping in mind user preferences and needs. Our research study was designed to inform evidence-based decision making around this issue through comparing the speed and accuracy of two popular point-of-care information tools. We designed and executed a cross-sectional research project which included the following phases: design, ethics approval, recruitment, experiment implementation, data analysis, and dissemination. **Outcomes:** We were ultimately successful in answering our research questions and publishing our study. Along the way, we faced challenges including recruitment of participants, bandwidth of study collaborators, and learning how to be effective researchers. Undertaking this work also led to many opportunities and lessons learned. **Discussion:** For most librarians, finding the time to conduct research projects can be intimidating, difficult, and often frustrating. Despite these challenges, research studies can help solve real problems in our profession. By sharing our experience, we hope to encourage attendees to take on these challenges. Let’s work together to build a more robust evidence base for making decisions in medical libraries. If we did it, you can too!

## CP10. CADTH Grey Matters, Search Filters, and PRESS Checklist Searching Tools: Modernizing Online Access

Monika Mierzwinski-Urban, David Kaunelis & Caitlyn Ford
Canadian Agency for Drugs and Technologies in Health

**Introduction:** With the objective of continual improvement, CADTH Research Information Services has committed to revitalizing their shared searching tools Grey Matters, Strings Attached Search Filters, and the Peer Review of Electronic Search Strategies (PRESS) Checklist. **Description:** Three CADTH tools: (1) Grey Matters checklist is a tool for finding health-related grey literature that is not commercially published. The checklist has been available online as a Word document for several years, both in English and French language. (2) CADTH creates pragmatically or systematically validated database search filters for use in evidence-based research, including those for retrieval of RCTs, systematic reviews, guidelines, and economic evaluations. Select CADTH filters have been available on our website for several years. However, the full range of our search filters has never been publicly accessible. (3) PRESS Checklist. **Outcomes:** A new online database is being developed for the CADTH Grey Matters checklist, via the ANDORNOT platform. This database will allow for regular checklist updates. It will also provide users with advanced search and browse options, record selection, and the ability to save or print selected results. A new database under development will house all CADTH filters, also using the ANDORNOT platform. In addition to the search strategy, detailed information on each filter will be searchable. The PRESS checklist will be modernized by the addition of an online version, which will include a fillable web form for all established PRESS checklist questions, with the ability to comment on the strategy. **Discussion:** These modernized CADTH search tools will aid evidence-based information retrieval.

## CP11. Reflecting and Adapting: Lessons Learned from Comparing Three Models of Program Delivery at a Distributed Medical School

Jackie Phinney & Robin Parker
Dalhousie University

**Introduction:** Since 2013, undergraduate medical (UGME) students at Dalhousie University's distributed medical program have participated in the Research in Medicine (RIM) program, requiring all students to complete research projects supervised by mentors. Library support for RIM has been present from its inception, most notably in the form of a yearly tutorial session on literature searching skills delivered to all first-year students across campuses, supplemented by an online asynchronous module and librarian consultations on demand. **Description:** The tutorial traditionally took place using the in-person model, but with the onset of the COVID-19 pandemic, the session shifted to online, synchronous delivery. Although we returned to the in-person format in 2021, changes in allotted curriculum time required us to revise our session again to fit into a shorter timeslot. **Outcomes:** After reviewing the respective quantitative and qualitative feedback from 2019, 2020, and 2021, we present the changes to content, mode of delivery, timing, and length of session that impacted students' receptiveness and satisfaction. We have continued to adjust the session to meet the changing needs of students and the UGME curriculum. **Discussion:** Student feedback from the past three years has provided valuable insight into what works in a literature searching skills tutorial within a mandatory research curriculum, what doesn't, and what factors are outside of our control as instructors. This program description will explore differences and similarities between the three delivery models and will encourage attendees to reflect on their own attempts to "try it again" in the classroom.

## CP12. Literature Searching for Ethics Reviews: State of the Search

Melissa Walter, Jennifer Horton, Amanda Hodgson
Canadian Agency for Drugs and Technologies in Health

**Introduction:** Systematic reviews with ethical analysis components are increasing in number, both as stand-alone reviews and as a component of health technology assessments. However, there is almost no guidance or information on how literature searches are performed for these reviews. We will analyze a large set of published systematic reviews with ethical analysis components in order to provide an overview of current literature searching practices in this field. **Methods:** As part of a larger project on literature searching methods for ethics reviews, we conducted a thorough search in six databases for a combination of the following three concepts: systematic reviews or HTAs, ethics, and literature searching or information retrieval. During our screening, we then compiled a set of roughly 350 published systematic reviews with ethical analysis components. We will extract data from these reviews on their literature search methods, such as: databases searched, grey literature sources searched, and search terms or search strategies used. **Results/Discussion:** Results and discussion forthcoming. **Conclusion:** Using the data from these reviews, we will be able to draw conclusions about the literature searching methods generally used in systematic reviews with ethical analysis components. This overview of current practice will help to inform future guidance in this field, as well as revealing areas where further research is needed.

## CP13. Health Sciences Librarian Participation in Continuing Education Initiatives: A Scoping Review

Jackie Phinney[1], Melissa Rothfus[1], Melissa Helwig[1] & Kristy Hancock[2]
Dalhousie University[1] & Maritime SPOR SUPPORT Unit[2]

**Introduction:** Continuing education (CE) is an expectation of many healthcare professionals and librarians to maintain credentials and keep their skills current. Since librarians have experience providing instruction, librarian provision of CE seems a natural fit. However, delivering content to healthcare professionals can be challenging due to time constraints, failed outreach, uncertainty surrounding topics, and more. Librarians may be wondering how they can provide helpful CE when they are unsure how to address these issues. **Description:** Mindful of these challenges, we began a scoping review in February 2020 (search updated September 2021) to examine the literature on librarian instruction of CE for healthcare professionals. We conducted a comprehensive search across seven databases to capture all published articles on this topic. We also performed forwards and backwards searching of included studies, solicited real-life teaching materials from colleagues, and searched for grey literature using Google. **Outcomes:** The literature revealed CE topics, delivery methods, and descriptions of initiatives. It also revealed gaps, such as lack of clear descriptions of assessment findings, efforts to accredit sessions, comprehensive learning objectives, mapping sessions to information literacy frameworks, and more. **Discussion:** Despite the challenges, creating and delivering instructional sessions is a valuable contribution that connects us with our users. Learning from the past can allow us to improve our instructional services. Gaps in those lessons remind us that we should evaluate, benchmark, and report our instruction so it remains resilient to the changing information landscape and allows us to build on the work of others.

## CP14. Getting Schooled on Instruction: Pedagogical Training Needs for Librarians

Catherine Pepper & T. Derek Halling
Texas A&M University

**Introduction**: This paper reports on results of a survey regarding librarians' training needs for instruction. While instruction is a competency recognized by national library associations and is a job requirement for many public services librarians, little opportunity for training in pedagogy exists in formal training venues, such as library school, resulting in librarians learning how to teach on their own. The research questions are: How do librarians learn to teach? What specific pedagogical skills do librarians need? What recommendations could lead to improved access to pedagogical training and to increased competency in instruction for librarians? **Method:** A 12-item survey on pedagogical training needs for health sciences and academic librarians will be sent to distribution lists of several library associations. Participants are asked to identify current teaching topics, how they learned to teach, and which of 38 areas of pedagogical training are needed. Quantitative items will be presented as categorical percentages and subgroup data will be reported using cross-tabulation analysis. Qualitative responses will be coded to derive themes. **Results/Conclusion**: Preliminary results from a small-sample (n=30) pilot survey indicated “Assessment methods”, “How to ensure deep learning rather than surface learning”, and “Curriculum and instructional development”” as areas of highest need. “Observing other instructors” and “On the job training” were the most frequently selected methods for pedagogical training, while “Courses in library school” received few selections. A strong need for more formal and accessible opportunities for gaining skills and knowledge in instruction was indicated. Final results from the full study will be rported.

## CP15. Let’s Try Something New: Launching the Toronto Health Libraries Association Library Technician SubCommittee

Alissa Epworth[1], Caleb Nault[2], Melissa Paladines[3], Micheal Reansbury[4] & Raluca Serban[2]
Evidence Partners[1] & University Health Network[2] & William Osler Health System[3] & Canadian College of Naturopathic Medicine[4]

**Introduction:** In 2020, the Toronto Health Libraries Association conducted a member engagement survey to determine the primary role of the chapter in supporting the health and hospital library workers of the greater Toronto area. Responses indicated the need to advocate for and offer targeted professional development to library technicians. In spring 2021, the association put out a call for volunteers and the Library Technician SubCommittee (LTSC) was launched. **Description:** The LTSC is composed of five library technicians with a mandate to produce a professional development program annually. The inaugural event was held in fall 2021 with the objectives of highlighting diverse and evolving roles for library technicians, recognizing opportunities, and developing skills to pursue those roles. In this session, we will describe the background context and processes involved in the creation and implementation of the LTSC. **Outcomes:** The online panel, “Emerging & Non-Traditional Roles for Library Technicians in 2021” was delivered to an audience of 60 participants from various professional backgrounds. Attendees were engaged and feedback has been extremely positive. A recording of the session has been viewed almost 60 times since the event. **Discussion:** Library technician roles are in flux. Existing professional development opportunities for technicians are scarce and need to be planned and delivered by library technicians for library technicians to meet our specific needs. We will discuss successes and lessons learned from the formation of the subcommittee and initial event and share future plans. The overwhelming success of the first event may indicate that technician-focused professional development should be given a national platform.

## CP16. Case Study: Scale of Librarian Involvement in Systematic Review Publications at University of Alberta

Megan Kennedy & Janice Kung
University of Alberta

**Introduction:** It is well documented that librarian involvement in systematic reviews generally increases quality of reporting and the review overall. We aim to analyze the level of librarian involvement in systematic reviews conducted at University of Alberta. **Methods:** Using Web of Science (WoS), we searched for systematic reviews completed in the past five years with a University of Alberta co-author. Systematic reviews identified through WoS will be screened in two phases: (1) Determining if the paper is a true systematic review publication (e.g. excluding duplicates, protocols, systematic review methodology papers, etc.), (2) Screening for librarian involvement (acknowledgement, co-author, or no involvement). **Results:** 640 reviews were analyzed for the following categories: (1) librarian named as a co-author; (2) librarian named in the acknowledgements section; (3) librarian mentioned in the body of the manuscript; (4) no librarian involvement. We identified 152 reviews who named a librarian as a co-author on the paper, 125 reviews that named a librarian in the acknowledgements section, and 268 reviews that mentioned a librarian in the body of the review. **Discussion:** There is a great deal of variation in how the work of librarians is reflected in systematic reviews. This was particularly apparent in reviews where a librarian was mentioned in the body of the review but they were not named as an author or formal acknowledgement. Continuing to educate researchers about the work of librarians is crucial to fully represent the value librarians bring to systematic reviews.

## CP17. "When You are Teaching to a Wall of Blank Screens": Lessons from Moving to Online Instruction in Hospital Libraries

Raluca Serban, Caleb Nault & Melanie Anderson
University Health Network

**Introduction:** Hospital health sciences libraries offer many in-person, single session workshops on topics ranging from database search skills to evidence based medicine to knowledge synthesis methods. These workshops need to compete with the extremely busy schedules of health professionals, students, administrative staff, and others. With many hospitals mandating staff work from home during the pandemic, it became imperative to engage our users virtually. We aimed to discover the practices that other hospital libraries developed around online instruction and launched a Quality Improvement project to inform and improve our own instruction practices. **Methods:** The Quality Improvement research methods used included a survey advertised to Canadian Hospital Libraries across relevant listservs, followed by a focus group with members of our own department. The survey included open-ended and multiple-choice questions and the focus group utilized a semi structured interview guide. **Results:** At the time of this submission we had 24 responses to our survey and are scheduling the focus group interviews. Results are forthcoming and will be updated. **Discussion:** In this session we will describe what the move to online instruction looked like in hospital library settings across Canada and within our own institution. We will explore and discuss the challenges, successes, lessons, and how these experiences fit in the larger picture of online instruction.

## CP18. Opening the Black Box of Librarian Instruction in Knowledge Synthesis Methods: Using Sociomaterial Theory to Explore Online Teaching

Robin Parker
Dalhousie University

**Introduction:** In addition to directly contributing search expertise to support knowledge synthesis (KS) teams, academic health librarians play significant roles training learners who are working on research reviews as part of their program or course work. While the skills development provided by librarians often centres around comprehensive searching, librarian expertise in KS methods more generally also benefits novice reviewers seeking assistance early in their research process. With the remote work and learning conditions brought by the COVID-19 pandemic much of this librarian instruction is conducted virtually, both to groups and individuals. A sociomaterial lens can illuminate the interconnected assemblages of human, material, technical, and textual components involved in online KS methods training, making visible the multifaceted techniques, skills, and knowledge that academic health librarians share with learners. **Methods:** Using my research as a worked example, I introduce the sociomateriality approach of Actor-Network Theory as applied to online teaching of KS methods in academic libraries. Through ethnographic research methods, including virtual focus groups, online teaching observations, and video-reflexive interviews, I trace the assemblages of human and non-human actors at play in these teaching encounters. **Results:** This preliminary report of an ongoing qualitative research project will focus on the role of theory in planning and conducting ethnographic research in a physically-distanced world. **Discussion:** Conducting research and delivering instruction in an online environment relies on complex assemblages of people, technology, physical resources, and textual documents. This project makes evident the richness and complexity of the often-invisible work of librarians teaching KS methods online.

## CP19. From a Service to a Partnership: Reimagining and Redesigning Knowledge Synthesis Work in an Academic Research Setting

Marisa Tippett, Maren Goodman, Meagan Stanley, Roxanne Isard, Christy Sich, Denise Horoky & Alanna Marson
Western University

**Introduction:** The pandemic has led to a boom in the volume of requests from students for systematic and scoping review assistance as well as requests from faculty for librarians to participate on systematic and scoping review research teams. A team of librarians at our large, research-intensive university saw the increase in demand for librarian participation on research teams as an opportunity to launch Partner with a Librarian, a 1-year pilot which connects faculty researchers with librarians who partner as co-authors in the planning, execution, and writing of knowledge syntheses**. Description:** Our systematic and scoping review team launched Partner with a Librarian in May 2021. Preparation for the launch included the creation of an application form, marketing materials, documents to assist the librarians conducting the reviews, and an assessment plan while navigating collective agreements and setting boundaries and expectations. **Outcomes:** Since inception, our team of six librarians have been research partners for twelve systematic and nine scoping reviews. Feedback has been overwhelmingly positive. Faculty reported that having a librarian on their research team improved their search strategy, significantly improved the research process, and our new initiative exceeded their expectations. **Discussion:** Performing searches for faculty is not new; however, formalizing this partnership and promoting the skills and expertise of the librarians on our library's Systematic and Scoping Review Team to our researchers was an excellent strategy to build strong relationships with our research community and raise the profile of the library. We are now in the process of securing additional resources so that we can convert this pilot project into a sustainable collaboration.

## CP20. Impact and Attention of Open Access COVID Research at Canadian Research-Intensive Universities

Shannon O'Reily[1], Jeffrey Demaine[2] & Mike Taylor[3]
Altmetric[1] & McMaster University[2] & Digital Science[3]

**Abstract:** Using the research output from McMaster University, we compare the citation impact and broader online engagement of Open Access (OA) versus non-OA ("closed") articles related to COVID-19. **Introduction:** In response to the COVID-19 pandemic, many publishers waived Article-Processing Fees, resulting in a surge of OA publications. We characterize the growth of this research field through the lens of NIH research categories, comparing global trends with Canada's 15 research-intensive universities ("The U15"). We then look at how growth in OA publications corresponded to an increase in COVID research engagement. **Methods:** A search was conducted using a structured search keyword or facet method in the Dimensions database. The results were partitioned by OA status, as represented by Dimensions and calculated by Unpaywall. The results set was passed into Altmetric to obtain social impact and public engagement data. **Results:** Using bibliometrics and alternative metrics, we saw an increase in attention around OA research articles versus closed articles. OA research has in particular been the subject of more interest in social media. The Altmetric attention score was also found to correlate with the number of academic citations - more citations equaled a higher Altmetric score. **Discussion:** OA articles are likely to have higher citations and broader engagement versus closed research. More research is required to see if this trend holds across research disciplines and beyond the special circumstances forged by COVID-19. Either way, it demonstrates the positive impact of OA research on academic and broader, societal engagement as it relates to COVID.

## CP21. Mind the gaps: opportunities for Consumer Health Provision by a Provincial Health Library System

Morgan Truax
Alberta Health Services

**Introduction:** To inform the design of a consumer health strategy for a provincial health library system, the library sought to understand patient challenges and barriers in accessing quality sources of health information. **Description:** Over a period of three months in the winter of 2020-2021, the library engaged in semi-structured interviews with relevant clinical and corporate stakeholders. Program leads were asked about their role in consumer health information provision and support, and where they saw gaps and opportunities within the organization. Answers were themed and analyzed, and an internal report was produced to guide next steps in developing a consumer health strategy. **Outcomes:** The environmental scan showed that there are clear challenges and barriers for patients in accessing quality health information. Barriers include the duration of interaction with a healthcare practitioner; the specificity of patient information needs; and the organizational emphasis on a single enterprise-wide patient information resource. More significant challenges include language; lack of health information literacy skills; and low digital literacy among certain populations. **Discussion:** The environmental scan results provided strong rationale for developing a cohesive consumer health strategy for the library. They highlighted valuable but disjointed resources and programs throughout the organization. A Consumer Health Framework was drafted to guide the library in setting achievable goals and leveraging existing supports. Due to the Covid-19 pandemic it was not possible to engage directly with patients for the initial scan, however consultation with patient advisory groups is now being planned to check and validate the library's direction.

## CP22. What Can be Learned from Failure? A Project on Predatory Journals

Amanda Ross-White & Dr. Rosemary Wilson
Queens University

**Introduction:** Predatory journals, with low standards of publication, mean flawed or fraudulent research can compromise future research. Often called “predatory” or “deceptive” publishers, both of these terms have an implication that the editors and publishers behind them have a motivation to deceive or con authors. However, the motivations remain an assumption because little is known about the individuals behind these journals. This research intended to use qualitative, in-depth interviews to find out more about the individuals behind predatory journals. By engaging with them directly, we hoped to gain an understanding of how they see themselves in the publishing landscape, what value they add and how they achieve these aims. **Methods:** E-mails received by the authors were mined for contact information of suspected predatory journals. Over the course of a year, 2804 e-mails were sent inviting respondents to an interview, for which there would be a small monetary compensation. **Results:** Despite sending 2804 e-mails, only three responses were received, and all three did not schedule an interview when prompted. Two of the three requested that a translator be present. A significant percentage of the e-mails (40.8%) bounced back, indicating the contact information was not valid. **Discussion:** While the information gained was limited, it would appear many are aware of the dubious nature of their journal and do not wish further scrutiny by being contacted. Others may lack the English language skills necessary to be engaged in basic written communication, let alone the more complex language and grammar of scientific publishing.

LT= Lightning Talks

## LT1. Guided Medline (Ovid) Exercise

Charlotte Beck & Kyle Gailing
University of British Columbia

**Introduction:** Library instruction in the health sciences includes searching Medline (Ovid). Our in-person teaching is supplemented with a locally developed Medline tutorial available on YouTube. With COVID-19 and exclusively doing remote teaching, we discovered that these tutorials provided insufficient guidance on using Medline (Ovid) to conduct a structured search. To fill this gap, we created The Guided Medline Exercise. **Description:** Using Libguides, we developed a learning object where each stage of the structured search is presented on its own page: analysis of the research question with PICO; finding and using subject headings and keywords for each concept; appropriate combining; use of limits; and saving and sharing the search and results. Each page includes an introduction; a step-by-step description, which is accompanied by a video; and a "build your own" section that guides the viewer through the process to develop a search on their specific topic. **Outcome:** The development of the learning object had various iterations that were informed by feedback from colleagues, student librarians, and graduate student researchers. A major enhancement to the design is the use of accordion boxes, which reduce clutter and reinforce the step-by-step process to develop a logical search. **Discussion:** Early feedback and observation indicates that this guided instruction, with an opportunity to practice, meets the objective to introduce Medline (Ovid), and to the process of creating a successful search. Further evaluation of its effectiveness will influence how it is disseminated.

### LT2. Celebrating Grey: Re-Developing an Author Recognition Program

Meredith Fischer
Wilfred Laurier University

**Introduction:** Author recognition programs are successful annual events at many academic and health sciences libraries. They provide an opportunity to identify, honour, and promote faculty publications, highlight library collections, and engage with the research community. While discussions about research assessment in tenure and promotion processes increasingly acknowledge the need to look beyond peer-reviewed publications, author recognition programs still tend to focus on traditional output such as books and journal articles. **Description:** Celebrating Laurier Achievements is a new iteration of a popular author recognition program at Wilfrid Laurier University, and a collaboration between the Library and the Office of Research Services. It has been re-developed to incorporate non-traditional output including grey literature such as reports, podcasts, and websites. This innovative program recognizes the scholarship and creativity of faculty, librarians, students, staff, alumni, and retirees with a launch event and an online exhibit in Omeka. **Outcomes:** Nearly 150 authors and creators on campus submitted their work to be included in Celebrating Laurier Achievements. The program committee (two librarians, a knowledge mobilization officer, an art gallery curator, and a sales and university press marketing coordinator) coordinated the design of promotional posters for each submission as well as the development of videos featuring 25 different achievements. These posters and videos were shared in the online exhibit, on YouTube, and through other social media platforms. **Discussion:** By expanding the scope of author recognition programs to include non-traditional output, libraries can acknowledge a greater diversity of achievements while offering more inclusive programming.

### LT3. Creating an Interdisciplinary Knowledge Synthesis Community of Practice at the University of Ottawa Library

Karine Fournier & Lindsey Sikora
University of Ottawa | Université d'Ottawa

With the growing demand for knowledge synthesis services across disciplines at the University of Ottawa Library, a Knowledge Synthesis Community of Practice (CoP) for all interested library employees was formed in 2021. This CoP gathers monthly with organized discussion and timely presentations with ideas put forward by its members. This talk will highlight how this CoP came together, its objectives and Terms of Reference, and the evolution of the topics of discussion that have arisen in order to ensure that members of the CoP can increase their competencies of knowledge synthesis.

### LT4. Active Learning in Action: Incorporating Virtual Escape Rooms and Scavenger Hunts in Library One-Shot Sessions

Julia Martyniuk
University of Toronto

Active learning is an important aspect in ensuring students' understanding and retention of instructional material. This presentation will review the successes and challenges of incorporating active learning opportunities in online one-shot instruction sessions, and focus on the role escape rooms and scavenger hunts play as an active learning technique. The case example of a specific one-shot library session, where medical graduate students searched for and accessed information and resources by way of a group escape room activity, will be highlighted. This presentation will underscore how escape rooms can be applied to orient students to academic libraries, thereby fostering engagement and interactivity when learning new content.

## LT5. Information Seeking Behavior of Early Career Family Physicians

Chidiebere Michael Iro & Joan C. Bartlett
McGill University

**Introduction:** Early career professionals experience transitions marked by changes in identities, roles, relationships, abilities and patterns of behavior. In addition to these, physicians at the beginning of their careers face the supplemental pressure of feeling that lives depend on the decisions they make at work, even as they navigate this transition. While residency offered immediate access to an experienced physician and free information resources, these are absent in established practice. Amongst family physicians, the most frequently arising information needs were questions related to diagnosis, treatment, and questions asked by patients. The most frequently consulted resources were colleagues, textbooks, journal or online publications, and medical applications. Inconsistent results were found for consults with librarians and information professionals. However, nothing was specific to the critical early phase of their career. Hence, this study explores the information needs and sources consulted by early career family physicians which influence their practice. **Methods:** An online survey will be distributed through the Facebook page for family physicians in their first five years of practice, with the appraisal of the Federation des Médecins Omnipraticienns du Quebec. Survey questions were informed by the literature review, and include questions related to demands at work that lead the physician to seek answers externally, as well as where they go to find these answers. **Results:** Survey distribution and data analysis are in progress and results will be presented. **Discussion:** Survey findings will be discussed, including implications for information professionals committed to information literacy in early career physicians.

## LT6. Searching Methods for Rapid Evidence Reviews During Public Health Emergencies

Leah Hagerman, Emily Clark, Dr. Sarah Neil-Sztramko, Taylor Colangeli & Dr. Maureen Dobbins
National Collaborating Centre for Methods and Tools

As COVID-19 related evidence surged early in the pandemic, numerous COVID specific databases and registries with varying features and functions emerged. Our team established a Rapid Evidence Service to conduct rapid reviews to inform the public health COVID-19 response. Here, we outline our lessons learned in searching for evidence related to the COVID-19 pandemic and provide recommendations for researchers and database developers when responding to public health emergencies. We evaluated databases and registries used to inform our rapid reviews based on three domains: topics and specialties (i.e., infection prevention and control, disease characteristics, surveillance and epidemiology, equity, policy, and mental health and substance use); level of evidence of indexed citations (i.e., systematic review, single studies, and expert opinion); and specific usability and functionality (i.e., advanced search features, search filters, citation exports, and citation sorting). We evaluated ten databases and four registries. Content and types of evidence included in each source varied. All databases included advanced search functionality; repositories had limited advanced search functionality. Features associated with reducing time and effort for conducting rapid reviews included advanced searching features, filters, sorting and export functions. Controlled vocabulary allowed us to efficiently identify the most relevant evidence in a timely manner; the ability to sort and filter evidence by title and abstract allowed us to reduce the search volume; and export functions allowed us to deduplicate results across databases and allowed for transparent screening methods. Our experiences can help inform future research strategies and the development and enhancement of new and previously existing evidence sources.

### LT7. Introducing our Library Champions

An initial attempt by the Library at Gloucestershire Hospitals NHS Trust, England to launch a Library Champion project was thwarted by Covid-19. The project relaunched, registering champions in July 2020, adapting the original plan to ensure it would not be detrimental to patient care. Twenty Champions were identified, who signed a contract formalizing their relationship with the library, receiving a Champions badge and quarterly update emails. Champions were drawn from different hospital settings (including Surgery, Critical Care, Infection Control, Dermatology, Estates, and Quality Improvement) and roles (including nurses, doctors, physio, psychologist, health care assistant, and managers). They committed to advocating the library service; signposting colleagues; promoting our services in their department; providing feedback and engagement on new or existing services; providing their opinion to improve services; and informing us of potential opportunities for involvement in events and projects. The Champion project was well received with engagement being recorded to measure impact, communication, and opportunities. We have seen an increase in inductions, tweets, and promotion of our services. We worked with champions to launch a Black History Month book challenge, a Brain Box for an offsite department and Get-Tea-Gethers. Due to interest, a 2022 cohort of Champions is planned.

### LT8. New Instructor Onboarding program for Medical Library Technicians

Ashley Jane Leonard & Jeanette Blanchard
Alberta Health Services

**Introduction:** Knowledge Resource Service (KRS) offers ten scheduled online information literacy courses. Until 2020, it was usual for these courses to be taught solely by librarians. Owing to staff shortages and Medical Library Technicians (MLT) expressing the desire to learn new skills, it was decided that MLTs would begin teaching two online courses. Five MLTs volunteered to take on instructional responsibilities. **Description:** Research was carried out to see what best practice dictated for in-house staff training in this area. Following this, an onboarding plan was created. This plan consisted of a 3-stage process which included observation, co-teaching, and solo-teaching. It was expected that librarian instructors would meet with their MLT co-instructor before their session to divide the content and practice the session. This onboarding program ran for approximately a year. **Outcomes:** The MLT instructors were surveyed following the completion of the onboarding process. Overall, the program was deemed a success: all MLTs have moved into solo teaching and have gained confidence in their teaching abilities. While not all co-teaching partnerships were considered successful, it was clear to see that in-house training is an effective way to up-skill KRS staff. **Discussion:** Some notable strengths of the program were skill development in online instruction and mentorship. The role of strong communication between co-instructors in ensuring positive teaching outcomes was a key take-away. The program was a success and we plan to use the same methods moving forward for new instructors.

### LT9. Can Artificial Intelligence Learn to Identify Systematic Reviews on the Effectiveness of Public Health Interventions?

Alanna Miller, Kristin Read, Heather Husson & Dr. Maureen Dobbins
The National Collaborating Centre for Methods and Tools

**Background:** Health Evidence™ aims to make it easier for public health decision-makers to use evidence in their practice. We provide access to over 7,700 critically appraised systematic reviews on the effectiveness of public health interventions. On average, over 13,000 records are screened each month for relevance to the registry. As the number of published reviews continues to grow each year, maintaining an up-to-date registry is increasingly resource intensive. Artificial intelligence (AI) may be one way to ensure maintenance of this registry continues to be feasible. **Methods:** The team uploaded a large training set (n=43,273) of records labelled as either relevant or not-relevant to DistillerSR and used the AI Preview and Rank function to predict the probability of relevance to the registry for each record. We identified an optimal threshold that correctly identifies the greatest number of not relevant records with minimal classification errors. We then tested this threshold on one year of manually screened records (n=89,832) for consistency and accuracy. **Results:** Health Evidence™ has successfully implemented the use of DistillerSR AI to support our monthly relevance screening. On average, after the removal of duplicate articles, the DistillerSR AI function identifies over 5,000 non-relevant articles each month, contributing to an 80% reduction in screening. This has reduced our manual screening burden and saves upwards of 20 hours of staff time per month! **Conclusion:** The use of AI shows promises to improve the feasibility of maintaining a large registry of quality appraised synthesis-level evidence relevant for public health decision making.

## LT10. Publishing for IMPACT: Publication Literacy Skills to Publish and Not Perish

Heather Cunningham, Graeme Slaght & Margaret Wall
University of Toronto

**Introduction:** Early-career researchers often learn about publishing through trial and error where publication and copyright decisions are made at the end of the research process, and few academic departments offer any form of structured training. The library saw an opportunity to develop a comprehensive and sustainable approach to address the publication literacy skills gap. **Description:** A five module asynchronous Getting Published Challenge was created in 2019 on the concepts of researcher identity, peer review, journal selection, scholarly sharing, and research impact. Hundreds of students have taken the Getting Published Challenge to build a personal Publication Plan. These modules were re-purposed as pre-work for a three-part Getting Published workshop series. The content of the workshops are divided into general information about the publishing landscape and copyright followed by discipline specific workshops on health sciences publishing and social science considerations. **Outcomes:** Having asynchronous modules as pre-work allows the synchronous Getting Published workshops to expand upon foundational concepts and allow for a dynamic and critical discourse on scholarly publishing and the decisions, impacts, and responsibility of authors. The modules and workshops combined have over-arching outcomes of developing an individual Publication Plan and re-positioning publication considerations to earlier in the research process. **Discussion:** The IMPACT acronym that is used as a framework for content delivery for the health sciences specific workshop will be discussed. The creation and delivery of publication workshops by an interdisciplinary team of copyright, health sciences, and social science librarians led to content and concepts that blended subject and functional expertise.

## LT11. Leveraging the Virtual Environment to Offer Cross-Institutional Workshops on Knowledge Synthesis Methodologies

Zahra Premji[1] & K. Alix Hayden[2]
University of Victoria[1] & University of Calgary[2]

**Introduction:** Workshops teaching knowledge synthesis (KS) methodologies are in high demand. However, teaching the how-to aspects of KS methodologies can require significant time commitment (6-8 hours) and expertise. Furthermore, due to the combined conceptual and technical content in KS workshops, co-teaching approaches are essential to facilitate instruction, especially in the virtual environment. Online workshop offerings have become common at many academic libraries since the pandemic, which has led to new opportunities to collaborate across institutions. **Description:** Two librarians, each from an academic library in different provinces, came together to virtually co-teach a 3-workshop series on KS methodologies. The in-depth workshops were piloted in fall 2021 and were offered to faculty and graduate students from any discipline, although the participants included a significant population from the health-related disciplines. The logistics and materials for all aspects of the workshop series were jointly developed and coordinated. **Outcomes and discussion:** The initial outcome of interest was the feasibility of offering these workshops jointly across institutions. Our pilot showed that challenges could be overcome with advanced planning, and the benefits of collaboratively offering the workshops outweighed the challenges. Offering these workshops as cross-institutional collaborations allows for expertise sharing and capacity building which benefits both institutions. Additional benefits of this approach include: potential cross-institution collaborations among participants, newer librarians learning to teach KS methodologies via co-teaching with a more experienced librarian, continuity and sustainability of the workshops. We envision these collaborative workshops being a regular virtual offering, and anticipate future collaborations with other libraries.

## LT12. "What a MeSH!" Improving Clinical Trial Retrieval and Record-Handling from the Cochrane Library

Alexandre Amar-Zifkin & Andrea Quaiattini
McGill University Health Centre

You need to search MeSH along with textwords when you're searching Cochrane, even if you're searching Medline in parallel, otherwise you might miss stuff. We'll explain why. Also, we will show you ONE WEIRD TRICK to get your citation manager to more completely ingest exports from Cochrane. It'll be fun (for certain values of fun).

## LT13. 3D Printing in Health Sciences Faculties and Libraries

Connie Winther, Laura Hamonic, Liz Dennett, Sandy Campbell
University of Alberta

**Introduction:** At the University of Alberta, we are currently in the planning stages for a new health sciences library. Three-dimensional (3D) printing is a technology of interest for the new library, building upon the success of the current 3D printing services already existing at the University of Alberta Library. 3D printing is used in teaching across the University and we were interested in determining what unique needs health sciences faculties have for 3D printing, and the role that the library has in fulfilling those needs. **Methods/Description:** An environmental scan is being completed to better understand the use of 3D printing and scanning in the health sciences. A narrative literature review will be completed to answer (1) What is the current state of 3D printing and scanning in health education in Canada? and (2) What is the current state of 3D printing and scanning within health science libraries? The focus of the literature review is on health sciences education, as we assumed that researchers requiring 3D printers would have access to their own printers. The results of the literature review will inform the questions that are asked in the environmental scan. **Results/Outcomes:** Preliminary findings from the literature review highlight that there are multiple reasons that 3D printing is used in health education including learning anatomy, procedural training, prototyping, and for producing other functional objects. **Discussion:** Additional findings will be shared during the presentation from the literature review and environmental scan.

### LT14. Local Authorship on Inuit Primary Health Research Publications

Connie Winther & Sandy Campbell
University of Alberta

**Background:** Inclusion of local and community researchers as integral members of research teams and, ultimately, as authors on research publications is one of the ways in which we can build equity and inclusion into research projects in Canada's North. This project examines the primary studies related to the health of Inuit people in Canada, to identify the frequency, extent and methods of the inclusion of local community members as authors. **Methods:** A title search of Ovid MEDLINE for articles about Inuit retrieved 472 articles. Studies that refer to primarily Inuit in Greenland and Alaska were excluded, as were those that were not primary studies of Inuit peoples' health. Samples of studies from 1999-2019 were reviewed for the inclusion of local community members as authors. Authorship was identified through author affiliation, author contribution statements or authorship references in the text of the articles. **Results:** Preliminary results show an increase in primary studies related to Inuit health over time and an increase in the recognition of local community engagement in research projects across Inuit Nunangat, the homeland of the Inuit in Canada. **Conclusions:** Over time, increasing numbers of acknowledgement indicate considerable community involvement in research. Over time, there is an increasing number of community co-authorships.

### LT15. Public Health Body Organizes Collaboration of Healthcare Librarians to Share and Peer Review COVID-19 Literature Searches During the Pandemic

Adam Tocock & Helene Gorring
Barts Health NHS Trust & Health Education England

Responding to the COVID-19 pandemic, Health Education England (HEE) mobilized a group of librarians in the NHS in England to inform the development of a platform for librarians to share COVID-19 search strategies and results. A COVID-19 Search Bank was quickly developed and hosted on the Knowledge for Healthcare website. It included searches' results, strategies, and keywords. The group wanted to take a pragmatic approach and to start sharing searches as quickly as possible, so a light-touch peer review was undertaken of searches and strategies submitted. This was an excellent example of collaborative working between NHS librarians. An evaluation of this initiative was carried out, with interviews of the librarians involved in its inception and peer reviewing, and a survey of NHS librarians in England. The key findings will be discussed.

### LT16. Instagram Stories @NOSMLibrary

**Introduction:** The Health Sciences Library at NOSM University has been reaching members on Instagram since 2017 and it is one of the library's most popular platforms. With the ongoing pandemic, the library aimed to utilize Instagram to its full potential to increase interaction with learners, faculty, and staff. The greatest concern was how time-consuming Instagram stories would be to produce. **Description:** With production time the initial primary concern, tools and resources readily available to the library were first assessed for their usefulness. Through trial and error over the course of the 2021-2022 academic year the Public Services Librarian and Library Assistant worked with these tools to develop an internal workflow**. Outcomes:** A variety of different story types were drafted in PowerPoint and produced in Instagram. This included library tours, bite size education, event promotion and more. Stories varied in length and time required to develop and produce. Ultimately a workflow was created to take the library's Instagram stories from the initial idea stage through to approval and production. Many tips and tricks and lessons were learned during this process. **Discussion:** The library's Instagram story workflow has streamlined the process of developing stories. Creating both longer and shorter stories enabled the library to keep Instagram story content fresh and scalable. Moving forward this workflow will be used to allow a variety of content experts in the library the opportunity to create story content.

PP=Poster Presentation

## PP1. Technology, Collection, and Budget Challenges of Growing Health Systems on Academic Health Science Libraries

Kelly Thormodson, Lori Cisney & Benjamin Hoover,
Pennsylvania State University

**Abstract:** The growth of health systems is having a major effect on academic libraries. The aim of this project is to survey academic health sciences libraries to learn about the challenges they face when they are part of a growing health system. In 2021, the Association of Academic Health Sciences Libraries (AAHSL) published a white paper on this issue, it did discuss the challenges of providing library services and resources to affiliated health systems, it did not describe in detail the specific challenges related to funding, collections, and technologies that this survey aims to do. **Introduction:** Previously published papers, have studied the relationships between academic health science libraries and hospitals, health systems and how to work with growing systems. However, they usually focus on best practices. These papers acknowledge the challenges in general but don't take time to acknowledge the specifics. This survey aims to help further understanding among academic health sciences libraries and librarians about the specific issues that are faced related to library collections, budget, and technology. **Methods:** A 15 question Qualtrics survey was sent to the AAHSL members' listserv. It was requested one completion per US based institution. It is a mixed methods survey with IRB approval. The survey was open for four weeks and there were 56 respondents. **Results:** The results of the survey can be divided into three categories: technology, budget, and collections, and politics & unique circumstances. There were many responses regarding current IT support and technology systems. Topics of concern included systems for assigning access (which included or excluded from resources) and authentication. In exploring collections funding and contracts for institutions with growing health systems, not surprisingly there were a wide variety of funding models expressed in the survey. In most cases funding comes from multiple sources, some expected and some unexpected). It was also common for the answer regarding who has access to resources to be complicated and these issues around access often bleed into the technical aspects of proxy and identity management. Challenges faced by the responding libraries often included comments around collections and budget, specifically reduced or flat budgets in the face of increasing resource costs and access issues tied to contract limitations. More than expected, libraries discussed the challenges of identifying appropriate charge back systems for obtaining funding from schools, colleges, and hospitals served along with the use of contracts and MOUs for designing relationships to who is and isn't served by the libraries in these new systems. **Discussion**: Technology: The two most common implementations of access control schemes for library resources were EZ Proxy (43%) and IP-ranges for access (34%). IT related responses also touched on complications from dealing with multiple systems and networks, the structure and number of IT groups involved, lack of identity management, communication and agency across entities, and known or suspected access issues not having immediate or simple solutions. Responders provided a spectrum of IT and technology related problems and solutions that were directly tied to the unique variables of culture, politics, and history of each institution/system. Budget & Collections: As expected many responses (15) identified funding coming from the University and many receive funding from the schools they serve, including Colleges/Schools of Medicine (11). Funding from hospitals and health systems was also a common response (14). Some surprises were instances where libraries receive funding from research grants received by the institution (2), direct government funding (4), and funding from foundations and non-profits (2). Five responding libraries discussed a need to track use of resources by type of user so that they could create a formula to share costs across schools, hospitals, and the health system. With regard to collections and a growing health system, the challenges seem to be relatively consistent and have to do with the cost of adding additional user groups and the difficulty in communicating this expense and need to administrators (7). Politics and unique circumstances: The survey asked if there was anything else they wanted the researchers to know. Many respondents discussed the MOUs and contracts between the libraries and the entities in the health systems. These arrangements covered what resources or services are provided, how costs are configured, and who has access. Politics and how that affected decision making were discussed, as well as the effects of the health system growth on the library resources that are directly connected to the Electronic Medical Record system.

## PP2. Teaching Them to Fish: A Structured Consultation Scheme for Teaching Synthesis Review Searching for Graduate Students in Nursing

Megan Kennedy
University of Alberta

This is a program description of a structured consultation scheme implemented to assist graduate students in the Faculty of Nursing who request librarian-performed searches for synthesis reviews. **Purpose:** In 2020, demand for librarian support for synthesis reviews exploded as human-subject research was suspended at post-secondary institutions due to COVID-19. Requests for mediated search support came from graduate students completing their thesis or dissertation work. Increasing demand necessitated prioritization of mediated searching services and the result was creating a consultation model specifically for graduate students requesting mediated search support. The aim was to improve students' confidence and self-sufficiency with advanced searching with only 2-3 hours of consultation with a librarian. **Methods:** The basic structure of the consultation scheme is as follows: Consultation 1: review protocol, select resources, develop search terms, develop Medline search; post consultation, student reviews search terms, first 100 results, and consults supervisor (aim to have this search finalized by next meeting). Consultation 2: translate search from Medline to other resources. Discuss search documentation and use of Covidence for screening; post consultation, student completes search translations and sends screenshots of searches to librarian for review. Consultation 3 (optional): discuss grey literature resources and documentation; address remaining search translation questions; post consultation, student searches completed**. Outcomes:** Feedback from students was positive. They appreciate learning more about the complexity of advanced searching and the unique experience to have such personalized guidance. Special attention must be given to students whose first language is not English as they may require additional support. **Discussion:** This approach has created capacity to be able to support more students using this method and also created capacity to perform mediated searches for faculty researchers.

## PP3. Let's Party! Celebrating National Medical Librarians Month!

Janice Thompson, Melissa Paladines & Anna Mann
William Osler Health System

**Introduction:** Over the past five years, William Osler Health System's Health Sciences Library has used the Medical Library Association's National Medical Librarians Month (NMLM), which occurs in October, as one of our main opportunities to promote our library. **Description:** Planning for our NMLM activities begins in June to allow time to obtain materials and design activities. There are one or two activities that repeat but our main activity is always new. In previous years, our activities have included open houses, displays, contests, and vendor swag giveaways. In 2021 due to the pandemic, our activities needed to be virtual to reach as many of our staff as possible. We chose Padlet, a fun and interactive tool to facilitate our contest and to gather library stories and experiences. We will highlight our activities with a focus on our latest Padlet contest, discuss the ways we promoted our activities and collaborated with our Communications department. **Outcomes:** Through our social media posts and displays in hospital high traffic areas, we were able to promote the library to our whole organization, reaching clinicians who may not be library users and raising awareness in our community. Our NMLM activities have enabled us to obtain feedback on our resources and services. **Discussion:** We will highlight our successes and share our disappointments through a lens of lessons learned. Our library team believes that celebrating NMLM is a great opportunity to raise awareness of our space, resources, and services.

## PP4. Five Years of Continuous Quality Improvement in a Rural and Remote Regional Health Library Service in British Columbia

**Introduction:** Employing the principles of continuous quality improvement, the library set out to improve its services, collections, and technology in order to increase access and use of evidence across a large geographical area. Historically, the library, with two full-time staff, had only served one hospital. After regionalization in the early 2000s, which added over 40 sites as customers, the library faced both technological and collections related barriers that needed addressing in order to improve service provisions outside its walls. **Description:** The librarian reviewed the current state of library services in 2015 to explore improvement opportunities. Data from two evaluation surveys, collection usage data and costs, environmental scans of other health authority libraries, and peer-reviewed evidence on health libraries were analyzed. Four goals were established in alignment with the organization's strategic plan for 2016-2021. Partners in the organization were engaged to leverage improvements in areas of evidence informed practice, awareness, orientations, and collections. **Outcomes:** Over 5 years, strategic incremental improvements led to a reduction in time-intensive tasks; increased usage of specific collections year-over-year; established metrics for refreshing and retiring collection materials and increased the ability of staff to access collections through significant technological change. **Discussion:** Setting specific goals aligned within the organization's strategic plan has allowed the library to flourish and become a more fulsome partner in the areas of practice standards development, quality improvement, and organizational learning. Leveraging technology for a regional approach also meant that when COVID-19 hit in the last year of the five year plan, the library saw almost no service disruptions during its brief ten week facility closure.

## PP5. Giving the Gift of Positivity to Our Students (and Ourselves): Running an Exam De-stressing Program

Glyneva Bradley-Ridout, Mikaela Mitchell, Jiewen (Jenny) Wu & Maggie Nevison
University of Toronto

**Introduction**: The Unstudy Spot is a program that aims to create fun, relaxing activities for students to enjoy while they take a break from studying. In December 2021, we ran our first in-person Unstudy Spot in two years. In this presentation, we will discuss our process of planning and executing the event at our health sciences library. **Description:** Planning began two months before the event. A working group was formed to brainstorm, distribute tasks, and select a theme. The event ran for a period of two weeks during the final exam period. Activities included a positivity wall, a scavenger hunt, snowflake making, and a collaborative jumbo crossword puzzle. The project team rotated responsibilities of re-stocking supplies and monitoring the space. We also incorporated a social media campaign. **Outcomes:** The most popular events were the positivity wall and snowflake making station. Many students also participated in the scavenger hunt, and the winners received a Starbucks gift card. Both students and organizers alike enjoyed the opportunity to safely engage with the physical library space again. **Discussion:** As health sciences librarians, we have a lot of demands on our time and increasingly large work portfolios. It can be difficult to find the time and energy for programming. However, we found that by creating and launching an event aimed at making students happy, we were able to bring some joy into our daily work as well. We hope to inspire colleagues to experiment with similar initiatives in their own spaces.

### PP6. How is Health Information Used? Lessons from Older Adults During the COVID-19 Pandemic

Xiaoqian Zhang & Joan Bartlett
McGill University

**Introduction:** The COVID-19 pandemic has had significant impacts on both how people manage their health, and how they are able to access information. As frequent users of healthcare services, older adults can benefit from health information. Health information can empower older adults, reduce their health risks, and support their health well-being. Previous studies have highlighted how older adults seek health information, but few have investigated the ultimate purpose of information-seeking, that is, how information is used. The objective of this research is to understand how older adults are using health information during the pandemic and the implications for the future. **Methods:** The pilot study is using semi-structured interviews with people aged 65 and above. The research defines information use as sets of actions people deal with obtained information, such as task performance, decision making, daily communication, and opinion changing. The interview questions will first focus on these actions. As information use is context-related and involves various cognitive and affective elements, the interview questions will also examine factors (e.g., context, thoughts, and feelings) related to older adults' health information use behaviour. Interviews will be analyzed using thematic analysis. **Results:** This pilot study is in progress. Preliminary results will be presented. **Discussion:** This will discuss the implications of the findings, including those relating to health librarians providing more targeted and practical intervention activities or information services to older adults in the post-pandemic era.

### PP7. Library as Hub for Digital Research and Learning: an Environmental Scan

Connie Winther, Lydia Zvyagintseva & Janice Kung
University of Alberta

**Introduction:** Digital tools and methods such as haptics and wearables (virtual, augmented, and extended reality), visualization and projection, virtual anatomy applications and displays, and media production equipment (sound booths, video creation kits, green screens) are increasingly used for teaching and research, but not often seen within Canadian health sciences libraries. **Description:** In order to guide the University of Alberta Library in the service planning and design of its new Health Sciences Library, we conducted an environmental scan with the following goals: (1) to plan the library's role in facilitating access to digital research technologies and services within the health sciences; (2) to understand contemporary services that support digital scholarship within health sciences libraries across North America; (3) to identify local and national communities of practice related to digital scholarship in health sciences libraries. The environmental scan consists of information gathering from three different environments. First, a scan of digital methods and practices used within the University of Alberta Health Sciences departments was conducted. Second, a narrative review of the literature was completed. Finally, a review of websites from health sciences academic libraries across North America was also carried out. **Outcomes:** We summarize the emergent themes in the literature and websites analyzed in this scan. Preliminary findings show that virtual reality is more common in health sciences libraries compared to other haptics and wearables. Virtual reality is used for curricular instruction and outreach, examples include virtual anatomy and pre-surgical case planning. Common challenges include finding sufficient room in the library for virtual reality, staff training, and software and device management.

## PP8. Discovering Our Collections: The Historical Development of Dentures

Maria Maddalena Zych & Usman Malik
University of Toronto

**Introduction:** Tooth loss impacts nutrition, diet, and socio-economic behaviours. Its prevalence makes it a global burden of health. Conventional dentures continue to be a common treatment due to their efficacy and low cost. They have improved nutrition and quality of life of patients throughout history. A scholarly exhibit describing this topic was lacking. Hence, this exhibit aims to: summarize materials and methods used to create dentures from ancient civilizations to early 19th century, and showcase the library's digital collection covering this topic. It can be accessed at https://exhibits.library.utoronto.ca/exhibits/show/history-dentures. **Description:** We surveyed the University of Toronto Dentistry Library's collection to learn about the historical development of dentures, and then created an online exhibition using the software OMEKA. The exhibition covers the developments of dentures during antiquity and the modern era, and describes the types of dentures, impression-taking methods, and denture material in the nineteenth century. The target audience is anyone interested in denture history. **Outcomes:** The key outcome for this program is to get users engaged with dentistry history, and specifically our library's rare book collections, which is digitized and made available for free on archive.org. An uptick in download and view metrics for the eBooks would affirm this program's effectiveness. **Discussion:** This exhibit will raise the public's interest in the history of science and the discoverability of the library's rare book collection. It will relay insights about denture history in an accessible and open manner. Future directions should consider other areas of health sciences' histories that would be useful to showcase.

## PP9. Revisiting the Current State of Systematic Review Support and Collaboration by Librarians at Canadian Universities

Catherine Boden
University of Saskatchewan

**Introduction:** Most Canadian academic libraries offer systematic review services. Initially these services supported researchers from primarily health and social sciences but, increasingly, those from a wider diversity of disciplines are seeking these services. This study updates and expands a similar study conducted in 2014 by surveying Canadian academic librarians from all disciplines about their current support for systematic reviews. **Methods:** A short (~15 minute) online questionnaire with Likert and dichotomous questions will be generated and distributed using Survey Monkey software. A convenience sample of Canadian academic librarians who have participated in a systematic review in the past 12 months will be recruited to participate via relevant librarian listservs. Data analysis will consist of calculating descriptive statistics, and tabulating short answer questions where an "other, please specify" option was given. **Results:** We anticipate distributing the survey in March 2022 and completing analysis by April 2022. **Conclusions:** This study will inform (1) understanding of the current landscape and how it has changed since 2014, and (2) provide evidence-based information to libraries about the current state of librarian involvement, including potential barriers, in providing systematic review support. Data availability statement: de-identified data will be openly available in a public repository that issues datasets with DOIs.

## PP10. A Case of De-duplication: Variations Among Four Different Methods

**Background:** De-duplication is a necessary but tedious component of literature searching. With ample methods available, it is difficult to know which are efficient and effective. Manual de-duplication is time consuming and can be challenging. Many information professionals are wary of automated processes due to lack of transparency. This case study compared four methods for de-duplication. **Methods:** We began with a set of 7,027 citations from a literature search conducted across six databases. This set was used to test de-duplication using four methods: manual de-duplication in EndNote; automated EndNote de-duplication using the Bramer method (Bramer et al. 2016); de-duplication in Covidence; and de-duplication in DistillerSR. We tracked number of duplicates removed. **Results:** Duplicates removed by each method ranged from 2,176 to 2254. Both screening softwares removed the fewest citations (Covidence-2,181; DistillerSR-2,176). Manual EndNote de-duplication removed 2,247, and the Bramer method removed the most citations, 2,254. **Discussion:** The range removed across the methods tested is small—78. Some limitations are that we opted for default selections in Covidence and DistillerSR. We also did not study the duplicates lists for accuracy, only comparing variation between tests. Lastly, our searches were strictly translated across databases—other translation approaches may yield different results. **Conclusion:** There are a variety of scenarios librarians encounter in de-duplicating large sets of results. Our case study displays the small amount of variation amongst the methods while dealing with a large set of results. Librarians and information specialists should discuss risks and advantages of these methods with their research teams.

JOURNAL INFORMATION
==============================
NLM Title Abbreviation: J Can Health Libr Assoc
Journal ID: J Can Health Libr Assoc
NLM Journal ID: 101229936
Journal ID: JCHLA

The Journal of the Canadian Health Libraries Association / Journal de l'Association des bibliothèques de la santé du Canada

EISSN: 1708-6892
Publisher: Canadian Health Libraries Association / Association des bibliotèques de la santé du Canada

ARTICLE INFORMATION
==============================
Subjects: IW = Interactive Workshop

IW1. Practical Guidance to Finding the Best Evidence During Public Health Emergencies

Mueller Mark 1
Askin Nicole 2
Brody Stacy 3
1 Saskatchewan Health Authority
2 University of Manitoba
3 George Washington University School of Medicine and Health Sciences

JOURNAL INFORMATION
==============================
NLM Title Abbreviation: J Can Health Libr Assoc
Journal ID: J Can Health Libr Assoc
NLM Journal ID: 101229936
Journal ID: JCHLA

The Journal of the Canadian Health Libraries Association / Journal de l'Association des bibliothèques de la santé du Canada

EISSN: 1708-6892
Publisher: Canadian Health Libraries Association / Association des bibliotèques de la santé du Canada

ARTICLE INFORMATION
==============================
Subjects: CP= Contributed Papers

CP1. The Right Tool, at the Right Time, for the Right Task: Information Management Platforms Used During the COVID-19 Pandemic

Sharma Minakshi 1
Maus Jeff 1
Ran Taiqi 1
Sabaliauskas Kelly 1
Xu Jielan 2
Yang Sabrena 1
Young Michael 3
Toronto Public Health Web Services Team 1
1 Toronto Public Health
2 City of Toronto
3 Toronto Paramedic Services

JOURNAL INFORMATION
==============================
NLM Title Abbreviation: J Can Health Libr Assoc
Journal ID: J Can Health Libr Assoc
NLM Journal ID: 101229936
Journal ID: JCHLA

The Journal of the Canadian Health Libraries Association / Journal de l'Association des bibliothèques de la santé du Canada

EISSN: 1708-6892
Publisher: Canadian Health Libraries Association / Association des bibliotèques de la santé du Canada

ARTICLE INFORMATION
==============================
Subjects: CP= Contributed Papers

CP2. From Silos to Synergy: Engaging in Interprofessional Collaboration during the COVID-19 Pandemic to Co-Create Information Dissemination Portals

Sharma Minakshi 1
Cheyne Jill 2
Corallo Ashley 1
Bianco Tracey Dal 1
Dearing-Vollett Julia 1
Liddy Ann 1
Pacht Chloe 1
Ran Taiqi 1
Seto Marisa 1
Toronto Public Health Web Services Team 1
Young Michael 3
1 Toronto Public Health
2 City of Toronto
3 Toronto Paramedic Services

JOURNAL INFORMATION
==============================
NLM Title Abbreviation: J Can Health Libr Assoc
Journal ID: J Can Health Libr Assoc
NLM Journal ID: 101229936
Journal ID: JCHLA

The Journal of the Canadian Health Libraries Association / Journal de l'Association des bibliothèques de la santé du Canada

EISSN: 1708-6892
Publisher: Canadian Health Libraries Association / Association des bibliotèques de la santé du Canada

ARTICLE INFORMATION
==============================
Subjects: CP= Contributed Papers

CP3. Experiences of Public Health Library Staff During the COVID-19 Pandemic: Stepping Up to Support Our Organizations and Reflecting on Lessons Learned

Faulkner Amy 1
Sharma Minakshi 2
Aulicino Maria 3
Harron Tanya 4
1 Simcoe Muskoka District Health Unit
2 Toronto Public Health
3 The Regional Municipality of York
4 Wellington-Dufferin-Guelph Public Health

JOURNAL INFORMATION
==============================
NLM Title Abbreviation: J Can Health Libr Assoc
Journal ID: J Can Health Libr Assoc
NLM Journal ID: 101229936
Journal ID: JCHLA

The Journal of the Canadian Health Libraries Association / Journal de l'Association des bibliothèques de la santé du Canada

EISSN: 1708-6892
Publisher: Canadian Health Libraries Association / Association des bibliotèques de la santé du Canada

ARTICLE INFORMATION
==============================
Subjects: CP= Contributed Papers

CP4. Urgent, Important, and Due ASAP: Providing Public Health Library Services in a Global Pandemic

Pach Beata
McArthur Allison
Kapetanos Domna
Skinner Hannah
Harker Lindsay
Massarella Susan
Public Health Ontario

JOURNAL INFORMATION
==============================
NLM Title Abbreviation: J Can Health Libr Assoc
Journal ID: J Can Health Libr Assoc
NLM Journal ID: 101229936
Journal ID: JCHLA

The Journal of the Canadian Health Libraries Association / Journal de l'Association des bibliothèques de la santé du Canada

EISSN: 1708-6892
Publisher: Canadian Health Libraries Association / Association des bibliotèques de la santé du Canada

ARTICLE INFORMATION
==============================
Subjects: CP= Contributed Papers

CP5. Reduce, Reuse, Repository: Creating a Hospital Poster Repository to Mitigate Research Waste, Mobilize and Preserve Institutional Knowledge

Osborne Zack
Myers Michael
Kishibe Teruko
Unity Health Toronto

JOURNAL INFORMATION
==============================
NLM Title Abbreviation: J Can Health Libr Assoc
Journal ID: J Can Health Libr Assoc
NLM Journal ID: 101229936
Journal ID: JCHLA

The Journal of the Canadian Health Libraries Association / Journal de l'Association des bibliothèques de la santé du Canada

EISSN: 1708-6892
Publisher: Canadian Health Libraries Association / Association des bibliotèques de la santé du Canada

ARTICLE INFORMATION
==============================
Subjects: CP= Contributed Papers

CP6. Novel Coronavirus, Novel Ideas: Collaborative Creation of a Mandatory Information Literacy Module for First-Year Health Sciences Students

Thorne Lydia
Ontario Tech University

JOURNAL INFORMATION
==============================
NLM Title Abbreviation: J Can Health Libr Assoc
Journal ID: J Can Health Libr Assoc
NLM Journal ID: 101229936
Journal ID: JCHLA

The Journal of the Canadian Health Libraries Association / Journal de l'Association des bibliothèques de la santé du Canada

EISSN: 1708-6892
Publisher: Canadian Health Libraries Association / Association des bibliotèques de la santé du Canada

ARTICLE INFORMATION
==============================
Subjects: CP= Contributed Papers

CP7. Information Seeking in the Time of COVID: How Do Young Adults Find COVID Related Information?

Bartlett Joan C.
Bowen-Ziecheck Aaron
Tsatas Sofie
McGill University

JOURNAL INFORMATION
==============================
NLM Title Abbreviation: J Can Health Libr Assoc
Journal ID: J Can Health Libr Assoc
NLM Journal ID: 101229936
Journal ID: JCHLA

The Journal of the Canadian Health Libraries Association / Journal de l'Association des bibliothèques de la santé du Canada

EISSN: 1708-6892
Publisher: Canadian Health Libraries Association / Association des bibliotèques de la santé du Canada

ARTICLE INFORMATION
==============================
Subjects: CP= Contributed Papers

CP8. Searches as Data: Using an Institutional Data Repository to Archive and Share Knowledge Synthesis Searches

Boruff Jill T.
Rod Alisa B.
McGill University

JOURNAL INFORMATION
==============================
NLM Title Abbreviation: J Can Health Libr Assoc
Journal ID: J Can Health Libr Assoc
NLM Journal ID: 101229936
Journal ID: JCHLA

The Journal of the Canadian Health Libraries Association / Journal de l'Association des bibliothèques de la santé du Canada

EISSN: 1708-6892
Publisher: Canadian Health Libraries Association / Association des bibliotèques de la santé du Canada

ARTICLE INFORMATION
==============================
Subjects: CP= Contributed Papers

CP9. Successes and Challenges of a 3 Year-Long Research Project: How We Did It and You Can Too

Bradley-Ridout Glyneva
Nekolaichuk Erica
Springall Elena
University of Toronto

JOURNAL INFORMATION
==============================
NLM Title Abbreviation: J Can Health Libr Assoc
Journal ID: J Can Health Libr Assoc
NLM Journal ID: 101229936
Journal ID: JCHLA

The Journal of the Canadian Health Libraries Association / Journal de l'Association des bibliothèques de la santé du Canada

EISSN: 1708-6892
Publisher: Canadian Health Libraries Association / Association des bibliotèques de la santé du Canada

ARTICLE INFORMATION
==============================
Subjects: CP= Contributed Papers

CP10. CADTH Grey Matters, Search Filters, and PRESS Checklist Searching Tools: Modernizing Online Access

Mierzwinski-Urban Monika
Kaunelis David
Ford Caitlyn
Canadian Agency for Drugs and Technologies in Health

JOURNAL INFORMATION
==============================
NLM Title Abbreviation: J Can Health Libr Assoc
Journal ID: J Can Health Libr Assoc
NLM Journal ID: 101229936
Journal ID: JCHLA

The Journal of the Canadian Health Libraries Association / Journal de l'Association des bibliothèques de la santé du Canada

EISSN: 1708-6892
Publisher: Canadian Health Libraries Association / Association des bibliotèques de la santé du Canada

ARTICLE INFORMATION
==============================
Subjects: CP= Contributed Papers

CP11. Reflecting and Adapting: Lessons Learned from Comparing Three Models of Program Delivery at a Distributed Medical School

Phinney Jackie
Parker Robin
Dalhousie University

JOURNAL INFORMATION
==============================
NLM Title Abbreviation: J Can Health Libr Assoc
Journal ID: J Can Health Libr Assoc
NLM Journal ID: 101229936
Journal ID: JCHLA

The Journal of the Canadian Health Libraries Association / Journal de l'Association des bibliothèques de la santé du Canada

EISSN: 1708-6892
Publisher: Canadian Health Libraries Association / Association des bibliotèques de la santé du Canada

ARTICLE INFORMATION
==============================
Subjects: CP= Contributed Papers

CP12. Literature Searching for Ethics Reviews: State of the Search

Walter Melissa
Horton Jennifer
Hodgson Amanda
Canadian Agency for Drugs and Technologies in Health

JOURNAL INFORMATION
==============================
NLM Title Abbreviation: J Can Health Libr Assoc
Journal ID: J Can Health Libr Assoc
NLM Journal ID: 101229936
Journal ID: JCHLA

The Journal of the Canadian Health Libraries Association / Journal de l'Association des bibliothèques de la santé du Canada

EISSN: 1708-6892
Publisher: Canadian Health Libraries Association / Association des bibliotèques de la santé du Canada

ARTICLE INFORMATION
==============================
Subjects: CP= Contributed Papers

CP13. Health Sciences Librarian Participation in Continuing Education Initiatives: A Scoping Review

Phinney Jackie 1
Rothfus Melissa 1
Helwig Melissa 1
Hancock Kristy 2
1 Dalhousie University
2 Maritime SPOR SUPPORT Unit

JOURNAL INFORMATION
==============================
NLM Title Abbreviation: J Can Health Libr Assoc
Journal ID: J Can Health Libr Assoc
NLM Journal ID: 101229936
Journal ID: JCHLA

The Journal of the Canadian Health Libraries Association / Journal de l'Association des bibliothèques de la santé du Canada

EISSN: 1708-6892
Publisher: Canadian Health Libraries Association / Association des bibliotèques de la santé du Canada

ARTICLE INFORMATION
==============================
Subjects: CP= Contributed Papers

CP14. Getting Schooled on Instruction: Pedagogical Training Needs for Librarians

Pepper Catherine
Halling T. Derek
Texas A&M University

JOURNAL INFORMATION
==============================
NLM Title Abbreviation: J Can Health Libr Assoc
Journal ID: J Can Health Libr Assoc
NLM Journal ID: 101229936
Journal ID: JCHLA

The Journal of the Canadian Health Libraries Association / Journal de l'Association des bibliothèques de la santé du Canada

EISSN: 1708-6892
Publisher: Canadian Health Libraries Association / Association des bibliotèques de la santé du Canada

ARTICLE INFORMATION
==============================
Subjects: CP= Contributed Papers

CP15. Let’s Try Something New: Launching the Toronto Health Libraries Association Library Technician SubCommittee

Epworth Alissa 1
Nault Caleb 2
Paladines Melissa 3
Reansbury Micheal 4
Serban Raluca 2
1 Evidence Partners
2 University Health Network
3 William Osler Health System
4 Canadian College of Naturopathic Medicine

JOURNAL INFORMATION
==============================
NLM Title Abbreviation: J Can Health Libr Assoc
Journal ID: J Can Health Libr Assoc
NLM Journal ID: 101229936
Journal ID: JCHLA

The Journal of the Canadian Health Libraries Association / Journal de l'Association des bibliothèques de la santé du Canada

EISSN: 1708-6892
Publisher: Canadian Health Libraries Association / Association des bibliotèques de la santé du Canada

ARTICLE INFORMATION
==============================
Subjects: CP= Contributed Papers

CP16. Case Study: Scale of Librarian Involvement in Systematic Review Publications at University of Alberta

Kennedy Megan
Kung Janice
University of Alberta

JOURNAL INFORMATION
==============================
NLM Title Abbreviation: J Can Health Libr Assoc
Journal ID: J Can Health Libr Assoc
NLM Journal ID: 101229936
Journal ID: JCHLA

The Journal of the Canadian Health Libraries Association / Journal de l'Association des bibliothèques de la santé du Canada

EISSN: 1708-6892
Publisher: Canadian Health Libraries Association / Association des bibliotèques de la santé du Canada

ARTICLE INFORMATION
==============================
Subjects: CP= Contributed Papers

CP17. “When You are Teaching to a Wall of Blank Screens”: Lessons from Moving to Online Instruction in Hospital Libraries

Serban Raluca
Nault Caleb
Anderson Melanie
University Health Network

JOURNAL INFORMATION
==============================
NLM Title Abbreviation: J Can Health Libr Assoc
Journal ID: J Can Health Libr Assoc
NLM Journal ID: 101229936
Journal ID: JCHLA

The Journal of the Canadian Health Libraries Association / Journal de l'Association des bibliothèques de la santé du Canada

EISSN: 1708-6892
Publisher: Canadian Health Libraries Association / Association des bibliotèques de la santé du Canada

ARTICLE INFORMATION
==============================
Subjects: CP= Contributed Papers

CP18. Opening the Black Box of Librarian Instruction in Knowledge Synthesis Methods: Using Sociomaterial Theory to Explore Online Teaching

Parker Robin
Dalhousie University

JOURNAL INFORMATION
==============================
NLM Title Abbreviation: J Can Health Libr Assoc
Journal ID: J Can Health Libr Assoc
NLM Journal ID: 101229936
Journal ID: JCHLA

The Journal of the Canadian Health Libraries Association / Journal de l'Association des bibliothèques de la santé du Canada

EISSN: 1708-6892
Publisher: Canadian Health Libraries Association / Association des bibliotèques de la santé du Canada

ARTICLE INFORMATION
==============================
Subjects: CP= Contributed Papers

CP19. From a Service to a Partnership: Reimagining and Redesigning Knowledge Synthesis Work in an Academic Research Setting

Tippett Marisa
Goodman Maren
Stanley Meagan
Isard Roxanne
Sich Christy
Horoky Denise
Marson Alanna
Western University

JOURNAL INFORMATION
==============================
NLM Title Abbreviation: J Can Health Libr Assoc
Journal ID: J Can Health Libr Assoc
NLM Journal ID: 101229936
Journal ID: JCHLA

The Journal of the Canadian Health Libraries Association / Journal de l'Association des bibliothèques de la santé du Canada

EISSN: 1708-6892
Publisher: Canadian Health Libraries Association / Association des bibliotèques de la santé du Canada

ARTICLE INFORMATION
==============================
Subjects: CP= Contributed Papers

CP20. Impact and Attention of Open Access COVID Research at Canadian Research-Intensive Universities

O’Reily Shannon 1
Demaine Jeffrey 2
Taylor Mike 3
1 Altmetric
2 McMaster University
3 Digital Science

JOURNAL INFORMATION
==============================
NLM Title Abbreviation: J Can Health Libr Assoc
Journal ID: J Can Health Libr Assoc
NLM Journal ID: 101229936
Journal ID: JCHLA

The Journal of the Canadian Health Libraries Association / Journal de l'Association des bibliothèques de la santé du Canada

EISSN: 1708-6892
Publisher: Canadian Health Libraries Association / Association des bibliotèques de la santé du Canada

ARTICLE INFORMATION
==============================
Subjects: CP= Contributed Papers

CP21. Mind the gaps: opportunities for Consumer Health Provision by a Provincial Health Library System

Truax Morgan
Alberta Health Services

JOURNAL INFORMATION
==============================
NLM Title Abbreviation: J Can Health Libr Assoc
Journal ID: J Can Health Libr Assoc
NLM Journal ID: 101229936
Journal ID: JCHLA

The Journal of the Canadian Health Libraries Association / Journal de l'Association des bibliothèques de la santé du Canada

EISSN: 1708-6892
Publisher: Canadian Health Libraries Association / Association des bibliotèques de la santé du Canada

ARTICLE INFORMATION
==============================
Subjects: CP= Contributed Papers

CP22. What Can be Learned from Failure? A Project on Predatory Journals

Ross-White Amanda
Wilson Rosemary Dr.
Queens University

JOURNAL INFORMATION
==============================
NLM Title Abbreviation: J Can Health Libr Assoc
Journal ID: J Can Health Libr Assoc
NLM Journal ID: 101229936
Journal ID: JCHLA

The Journal of the Canadian Health Libraries Association / Journal de l'Association des bibliothèques de la santé du Canada

EISSN: 1708-6892
Publisher: Canadian Health Libraries Association / Association des bibliotèques de la santé du Canada

ARTICLE INFORMATION
==============================
Subjects: LT= Lightning Talks

LT1. Guided Medline (Ovid) Exercise

Beck Charlotte
Gailing Kyle
University of British Columbia

JOURNAL INFORMATION
==============================
NLM Title Abbreviation: J Can Health Libr Assoc
Journal ID: J Can Health Libr Assoc
NLM Journal ID: 101229936
Journal ID: JCHLA

The Journal of the Canadian Health Libraries Association / Journal de l'Association des bibliothèques de la santé du Canada

EISSN: 1708-6892
Publisher: Canadian Health Libraries Association / Association des bibliotèques de la santé du Canada

ARTICLE INFORMATION
==============================
Subjects: LT= Lightning Talks

LT2. Celebrating Grey: Re -Developing an Author Recognition Program

Fischer Meredith
Wilfred Laurier University

JOURNAL INFORMATION
==============================
NLM Title Abbreviation: J Can Health Libr Assoc
Journal ID: J Can Health Libr Assoc
NLM Journal ID: 101229936
Journal ID: JCHLA

The Journal of the Canadian Health Libraries Association / Journal de l'Association des bibliothèques de la santé du Canada

EISSN: 1708-6892
Publisher: Canadian Health Libraries Association / Association des bibliotèques de la santé du Canada

ARTICLE INFORMATION
==============================
Subjects: LT= Lightning Talks

LT3. Creating an Interdisciplinary Knowledge Synthesis Community of Practice at the University of Ottawa Library

Fournier Karine
Sikora Lindsey
University of Ottawa | Université d'Ottawa

JOURNAL INFORMATION
==============================
NLM Title Abbreviation: J Can Health Libr Assoc
Journal ID: J Can Health Libr Assoc
NLM Journal ID: 101229936
Journal ID: JCHLA

The Journal of the Canadian Health Libraries Association / Journal de l'Association des bibliothèques de la santé du Canada

EISSN: 1708-6892
Publisher: Canadian Health Libraries Association / Association des bibliotèques de la santé du Canada

ARTICLE INFORMATION
==============================
Subjects: LT= Lightning Talks

LT4. Active Learning in Action: Incorporating Virtual Escape Rooms and Scavenger Hunts in Library One-Shot Sessions

Martyniuk Julia
University of Toronto

JOURNAL INFORMATION
==============================
NLM Title Abbreviation: J Can Health Libr Assoc
Journal ID: J Can Health Libr Assoc
NLM Journal ID: 101229936
Journal ID: JCHLA

The Journal of the Canadian Health Libraries Association / Journal de l'Association des bibliothèques de la santé du Canada

EISSN: 1708-6892
Publisher: Canadian Health Libraries Association / Association des bibliotèques de la santé du Canada

ARTICLE INFORMATION
==============================
Subjects: LT= Lightning Talks

LT5. Information Seeking Behavior of Early Career Family Physicians

Iro Chidiebere Michael
Bartlett Joan C.
McGill University

JOURNAL INFORMATION
==============================
NLM Title Abbreviation: J Can Health Libr Assoc
Journal ID: J Can Health Libr Assoc
NLM Journal ID: 101229936
Journal ID: JCHLA

The Journal of the Canadian Health Libraries Association / Journal de l'Association des bibliothèques de la santé du Canada

EISSN: 1708-6892
Publisher: Canadian Health Libraries Association / Association des bibliotèques de la santé du Canada

ARTICLE INFORMATION
==============================
Subjects: LT= Lightning Talks

LT6. Searching Methods for Rapid Evidence Reviews During Public Health Emergencies

Hagerman Leah
Clark Emily
Neil-Sztramko Sarah Dr.
Colangeli Taylor
Dobbins Maureen Dr.
National Collaborating Centre for Methods and Tools

JOURNAL INFORMATION
==============================
NLM Title Abbreviation: J Can Health Libr Assoc
Journal ID: J Can Health Libr Assoc
NLM Journal ID: 101229936
Journal ID: JCHLA

The Journal of the Canadian Health Libraries Association / Journal de l'Association des bibliothèques de la santé du Canada

EISSN: 1708-6892
Publisher: Canadian Health Libraries Association / Association des bibliotèques de la santé du Canada

ARTICLE INFORMATION
==============================
Subjects: LT= Lightning Talks

LT7. Introducing our Library Champions

George Chloe
Gloucestershire Hospitals NHS foundation Trust

JOURNAL INFORMATION
==============================
NLM Title Abbreviation: J Can Health Libr Assoc
Journal ID: J Can Health Libr Assoc
NLM Journal ID: 101229936
Journal ID: JCHLA

The Journal of the Canadian Health Libraries Association / Journal de l'Association des bibliothèques de la santé du Canada

EISSN: 1708-6892
Publisher: Canadian Health Libraries Association / Association des bibliotèques de la santé du Canada

ARTICLE INFORMATION
==============================
Subjects: LT= Lightning Talks

LT8. New Instructor Onboarding program for Medical Library Technicians

Leonard Ashley Jane
Blanchard Jeanette
Alberta Health Services

JOURNAL INFORMATION
==============================
NLM Title Abbreviation: J Can Health Libr Assoc
Journal ID: J Can Health Libr Assoc
NLM Journal ID: 101229936
Journal ID: JCHLA

The Journal of the Canadian Health Libraries Association / Journal de l'Association des bibliothèques de la santé du Canada

EISSN: 1708-6892
Publisher: Canadian Health Libraries Association / Association des bibliotèques de la santé du Canada

ARTICLE INFORMATION
==============================
Subjects: LT= Lightning Talks

LT9. Can Artificial Intelligence Learn to Identify Systematic Reviews on the Effectiveness of Public Health Interventions?

Miller Alanna
Read Kristin
Husson Heather
Dobbins Maureen Dr.
The National Collaborating Centre for Methods and Tools

JOURNAL INFORMATION
==============================
NLM Title Abbreviation: J Can Health Libr Assoc
Journal ID: J Can Health Libr Assoc
NLM Journal ID: 101229936
Journal ID: JCHLA

The Journal of the Canadian Health Libraries Association / Journal de l'Association des bibliothèques de la santé du Canada

EISSN: 1708-6892
Publisher: Canadian Health Libraries Association / Association des bibliotèques de la santé du Canada

ARTICLE INFORMATION
==============================
Subjects: LT= Lightning Talks

LT10. Publishing for IMPACT: Publication Literacy Skills to Publish and Not Perish

Cunningham Heather
Slaght Graeme
Wall Margaret
University of Toronto

JOURNAL INFORMATION
==============================
NLM Title Abbreviation: J Can Health Libr Assoc
Journal ID: J Can Health Libr Assoc
NLM Journal ID: 101229936
Journal ID: JCHLA

The Journal of the Canadian Health Libraries Association / Journal de l'Association des bibliothèques de la santé du Canada

EISSN: 1708-6892
Publisher: Canadian Health Libraries Association / Association des bibliotèques de la santé du Canada

ARTICLE INFORMATION
==============================
Subjects: LT= Lightning Talks

LT11. Leveraging the Virtual Environment to Offer Cross-Institutional Workshops on Knowledge Synthesis Methodologies

Premji Zahra 1
Hayden K. Alix 2
1 University of Victoria
2 University of Calgary

JOURNAL INFORMATION
==============================
NLM Title Abbreviation: J Can Health Libr Assoc
Journal ID: J Can Health Libr Assoc
NLM Journal ID: 101229936
Journal ID: JCHLA

The Journal of the Canadian Health Libraries Association / Journal de l'Association des bibliothèques de la santé du Canada

EISSN: 1708-6892
Publisher: Canadian Health Libraries Association / Association des bibliotèques de la santé du Canada

ARTICLE INFORMATION
==============================
Subjects: LT= Lightning Talks

LT12. “What a MeSH!” Improving Clinical Trial Retrieval and Record-Handling from the Cochrane Library

Amar-Zifkin Alexandre
Quaiattini Andrea
McGill University Health Centre

JOURNAL INFORMATION
==============================
NLM Title Abbreviation: J Can Health Libr Assoc
Journal ID: J Can Health Libr Assoc
NLM Journal ID: 101229936
Journal ID: JCHLA

The Journal of the Canadian Health Libraries Association / Journal de l'Association des bibliothèques de la santé du Canada

EISSN: 1708-6892
Publisher: Canadian Health Libraries Association / Association des bibliotèques de la santé du Canada

ARTICLE INFORMATION
==============================
Subjects: LT= Lightning Talks

LT13. 3D Printing in Health Sciences Faculties and Libraries

Winther Connie
Hamonic Laura
Dennett Liz
Campbell Sandy
University of Alberta

JOURNAL INFORMATION
==============================
NLM Title Abbreviation: J Can Health Libr Assoc
Journal ID: J Can Health Libr Assoc
NLM Journal ID: 101229936
Journal ID: JCHLA

The Journal of the Canadian Health Libraries Association / Journal de l'Association des bibliothèques de la santé du Canada

EISSN: 1708-6892
Publisher: Canadian Health Libraries Association / Association des bibliotèques de la santé du Canada

ARTICLE INFORMATION
==============================
Subjects: LT= Lightning Talks

LT14. Local Authorship on Inuit Primary Health Research Publications

Winther Connie
Campbell Sandy
University of Alberta

JOURNAL INFORMATION
==============================
NLM Title Abbreviation: J Can Health Libr Assoc
Journal ID: J Can Health Libr Assoc
NLM Journal ID: 101229936
Journal ID: JCHLA

The Journal of the Canadian Health Libraries Association / Journal de l'Association des bibliothèques de la santé du Canada

EISSN: 1708-6892
Publisher: Canadian Health Libraries Association / Association des bibliotèques de la santé du Canada

ARTICLE INFORMATION
==============================
Subjects: LT= Lightning Talks

LT15. Public Health Body Organizes Collaboration of Healthcare Librarians to Share and Peer Review COVID-19 Literature Searches During the Pandemic

Tocock Adam
Gorring Helene
Barts Health NHS Trust & Health Education England

JOURNAL INFORMATION
==============================
NLM Title Abbreviation: J Can Health Libr Assoc
Journal ID: J Can Health Libr Assoc
NLM Journal ID: 101229936
Journal ID: JCHLA

The Journal of the Canadian Health Libraries Association / Journal de l'Association des bibliothèques de la santé du Canada

EISSN: 1708-6892
Publisher: Canadian Health Libraries Association / Association des bibliotèques de la santé du Canada

ARTICLE INFORMATION
==============================
Subjects: LT= Lightning Talks

LT16. Instagram Stories @NOSMLibrary

Campbell Alanna
Northern Ontario School of Medicine

JOURNAL INFORMATION
==============================
NLM Title Abbreviation: J Can Health Libr Assoc
Journal ID: J Can Health Libr Assoc
NLM Journal ID: 101229936
Journal ID: JCHLA

The Journal of the Canadian Health Libraries Association / Journal de l'Association des bibliothèques de la santé du Canada

EISSN: 1708-6892
Publisher: Canadian Health Libraries Association / Association des bibliotèques de la santé du Canada

ARTICLE INFORMATION
==============================
Subjects: PP=Poster Presentation

PP1. Technology, Collection, and Budget Challenges of Growing Health Systems on Academic Health Science Libraries

Thormodson Kelly
Cisney Lori
Hoover Benjamin
Pennsylvania State University

JOURNAL INFORMATION
==============================
NLM Title Abbreviation: J Can Health Libr Assoc
Journal ID: J Can Health Libr Assoc
NLM Journal ID: 101229936
Journal ID: JCHLA

The Journal of the Canadian Health Libraries Association / Journal de l'Association des bibliothèques de la santé du Canada

EISSN: 1708-6892
Publisher: Canadian Health Libraries Association / Association des bibliotèques de la santé du Canada

ARTICLE INFORMATION
==============================
Subjects: PP=Poster Presentation

PP2. Teaching Them to Fish: A Structured Consultation Scheme for Teaching Synthesis Review Searching for Graduate Students in Nursing

Kennedy Megan
University of Alberta

JOURNAL INFORMATION
==============================
NLM Title Abbreviation: J Can Health Libr Assoc
Journal ID: J Can Health Libr Assoc
NLM Journal ID: 101229936
Journal ID: JCHLA

The Journal of the Canadian Health Libraries Association / Journal de l'Association des bibliothèques de la santé du Canada

EISSN: 1708-6892
Publisher: Canadian Health Libraries Association / Association des bibliotèques de la santé du Canada

ARTICLE INFORMATION
==============================
Subjects: PP=Poster Presentation

PP3. Let's Party! Celebrating National Medical Librarians Month!

Thompson Janice
Paladines Melissa
Mann Anna
William Osler Health System

JOURNAL INFORMATION
==============================
NLM Title Abbreviation: J Can Health Libr Assoc
Journal ID: J Can Health Libr Assoc
NLM Journal ID: 101229936
Journal ID: JCHLA

The Journal of the Canadian Health Libraries Association / Journal de l'Association des bibliothèques de la santé du Canada

EISSN: 1708-6892
Publisher: Canadian Health Libraries Association / Association des bibliotèques de la santé du Canada

ARTICLE INFORMATION
==============================
Subjects: PP=Poster Presentation

PP4. Five Years of Continuous Quality Improvement in a Rural and Remote Regional Health Library Service in British Columbia

Creaser Julie
Northern Health Library Services

JOURNAL INFORMATION
==============================
NLM Title Abbreviation: J Can Health Libr Assoc
Journal ID: J Can Health Libr Assoc
NLM Journal ID: 101229936
Journal ID: JCHLA

The Journal of the Canadian Health Libraries Association / Journal de l'Association des bibliothèques de la santé du Canada

EISSN: 1708-6892
Publisher: Canadian Health Libraries Association / Association des bibliotèques de la santé du Canada

ARTICLE INFORMATION
==============================
Subjects: PP=Poster Presentation

PP5. Giving the Gift of Positivity to Our Students (and Ourselves): Running an Exam De stressing Program

Bradley-Ridout Glyneva
Mitchell Mikaela
Wu Jiewen (Jenny)
Nevison Maggie
University of Toronto

JOURNAL INFORMATION
==============================
NLM Title Abbreviation: J Can Health Libr Assoc
Journal ID: J Can Health Libr Assoc
NLM Journal ID: 101229936
Journal ID: JCHLA

The Journal of the Canadian Health Libraries Association / Journal de l'Association des bibliothèques de la santé du Canada

EISSN: 1708-6892
Publisher: Canadian Health Libraries Association / Association des bibliotèques de la santé du Canada

ARTICLE INFORMATION
==============================
Subjects: PP=Poster Presentation

PP6. How is Health Information Used? Lessons from Older Adults During the COVID-19 Pandemic

Zhang Xiaoqian
Bartlett Joan
McGill University

JOURNAL INFORMATION
==============================
NLM Title Abbreviation: J Can Health Libr Assoc
Journal ID: J Can Health Libr Assoc
NLM Journal ID: 101229936
Journal ID: JCHLA

The Journal of the Canadian Health Libraries Association / Journal de l'Association des bibliothèques de la santé du Canada

EISSN: 1708-6892
Publisher: Canadian Health Libraries Association / Association des bibliotèques de la santé du Canada

ARTICLE INFORMATION
==============================
Subjects: PP=Poster Presentation

PP7. Library as Hub for Digital Research and Learning: an Environmental Scan

Winther Connie
Zvyagintseva Lydia
Kung Janice
University of Alberta

JOURNAL INFORMATION
==============================
NLM Title Abbreviation: J Can Health Libr Assoc
Journal ID: J Can Health Libr Assoc
NLM Journal ID: 101229936
Journal ID: JCHLA

The Journal of the Canadian Health Libraries Association / Journal de l'Association des bibliothèques de la santé du Canada

EISSN: 1708-6892
Publisher: Canadian Health Libraries Association / Association des bibliotèques de la santé du Canada

ARTICLE INFORMATION
==============================
Subjects: PP=Poster Presentation

PP8. Discovering Our Collections: The Historical Development of Dentures

Zych Maria Maddalena
Malik Usman
University of Toronto

JOURNAL INFORMATION
==============================
NLM Title Abbreviation: J Can Health Libr Assoc
Journal ID: J Can Health Libr Assoc
NLM Journal ID: 101229936
Journal ID: JCHLA

The Journal of the Canadian Health Libraries Association / Journal de l'Association des bibliothèques de la santé du Canada

EISSN: 1708-6892
Publisher: Canadian Health Libraries Association / Association des bibliotèques de la santé du Canada

ARTICLE INFORMATION
==============================
Subjects: PP=Poster Presentation

PP9. Revisiting the Current State of Systematic Review Support and Collaboration by Librarians at Canadian Universities

Boden Catherine
University of Saskatchewan

JOURNAL INFORMATION
==============================
NLM Title Abbreviation: J Can Health Libr Assoc
Journal ID: J Can Health Libr Assoc
NLM Journal ID: 101229936
Journal ID: JCHLA

The Journal of the Canadian Health Libraries Association / Journal de l'Association des bibliothèques de la santé du Canada

EISSN: 1708-6892
Publisher: Canadian Health Libraries Association / Association des bibliotèques de la santé du Canada

ARTICLE INFORMATION
==============================
Subjects: PP=Poster Presentation

PP10. A Case of De-duplication: Variations Among Four Different Methods

Horton Jennifer
Canadian Agency for Drugs and Technologies in Health

